# The tumor suppressor interferon regulatory factor 8 inhibits β-catenin signaling in breast cancers, but is frequently silenced by promoter methylation

**DOI:** 10.18632/oncotarget.16511

**Published:** 2017-03-23

**Authors:** Xinrong Luo, Xin Xiong, Qing Shao, Tingxiu Xiang, Lili Li, Xuedong Yin, Xia Li, Qian Tao, Guosheng Ren

**Affiliations:** ^1^ Chongqing Key Laboratory of Molecular Oncology and Epigenetics, The First Affiliated Hospital of Chongqing Medical University, Chongqing, China; ^2^ Department of Endocrine and Breast Surgery, The First Affiliated Hospital of Chongqing Medical University, Chongqing, China; ^3^ Cancer Epigenetics Laboratory, Department of Clinical Oncology, State Key Laboratory of Oncology in South China, Sir YK Pao Center for Cancer and Li Ka Shine Institute of Health Sciences, The Chinese University of Hong Kong and CUHK Shenzhen Research Institute, Shatin, Hong Kong

**Keywords:** interferon regulatory factor 8, methylation, tumor suppressor, breast cancer, β-catenin

## Abstract

Interferon (IFN) regulatory factor 8 is encoded by a novel candidate tumor suppressor gene (*IRF8*), its promotor is frequently methylated in multiple cancers. However, the promoter methylation status, functions and underlying mechanisms of *IRF8* in breast cancer remain unclear. We found that *IRF8* was downregulated in breast cancer cell lines and primary tumors, compared with normal breast tissues, mainly because of aberrant promoter methylation. However, its expression was not associated with pathological characteristics. Restoration of *IRF8* expression suppressed cell proliferation, colony formation, 5-ethynyl-2′-deoxyuridine incorporation, cell migration and invasion, and induced apoptosis and cell cycle arrest *in vitro*. IRF8 also inhibited xenograft growth in nude mice *in vivo*. Competition with *IRF8* function by *IRF8* mutant (K79E) enhanced cell migration and invasion in 4T1 murine cells *in vitro*. Importantly, *IRF8*, as both downstream target gene and regulator of IFN-γ/STAT1 signaling, inhibited canonical β-catenin signaling. These findings identify IRF8 as a novel tumor suppressor regulating IFN-γ/STAT1 signaling and β-catenin signaling in breast cancer.

## INTRODUCTION

Breast cancer remains the most common female malignancy, despite progress in diagnostic techniques and multimodality therapy [1, 2]. Oncogene activation and tumor suppressor gene (TSG) inactivation are major molecular events responsible for transforming normal mammary epithelia into tumorous epithelia [3–5]. Increasing evidences suggest that suppression of candidate TSG expression in many cancers, including breast cancer, is mainly the result of promoter CpG methylation [6–8]. It is therefore crucial to understand the epigenetic inactivation of novel TSGs in breast cancers.

Interferon (IFN) regulatory factor 8 (*IRF8*, also named IFN consensus sequence binding protein, *ICSBP*) is an IRF family transcription factor located at chromosomal region 16q24.1 [9]. IRF8 has been validated as a downstream target of the IFN-γ/signal transducer and activator of transcription 1 (STAT1) signaling pathway [10]. We previously showed that *IRF8* expression was inversely correlated with hypermethylation of its promoter region in various cancer cell types, including breast cancer cells [11]. Moreover, the promoter of *IRF8* has been shown to be methylated in gastric, colon, and lung carcinomas, and in myelogenous leukemia and multiple myeloma [12–16]. Furthermore, disruption of IRF8 function reduced tumor cell sensitivity to apoptosis and increased their metastatic potential, indicating its role as a TSG in multiple cancers [10, 17]. Although some evidence suggests that IRF8 enhanced cell proliferation, motility and invasion via TGF-β signaling [18, 19], its function and underlying mechanism in breast cancer remain unclear.

Wnt/β-catenin signaling pathways are involved in cell proliferation, stem cell characteristics, migration, and metastasis, and are activated in numerous cancers, including breast cancers [3, 20, 21]. Several studies have shown a negative correlation between IRF8 and β-catenin signaling in myeloid cells and leukemia [22, 23], indicating that IRF8 may at least partly suppress β-catenin signaling in solid tumors.

This study showed that the *IRF8* promoter was frequently hypermethylated in primary breast cancers, and IRF8 served as a main downstream factor in the IFN-γ/STAT1 signaling pathway, with a possible role in enhancing the anti-tumor effect of IFN-γ. Furthermore, *IRF8* acted as a candidate TSG in breast cancer, at least partly by suppressing the β-catenin signaling pathway. These results indicate that *IRF8* serves as a candidate TSG that is frequently hypermethylated in breast cancers.

## RESULTS

### IRF8 is an independent prognostic factor for breast cancer

**Figure 1:**
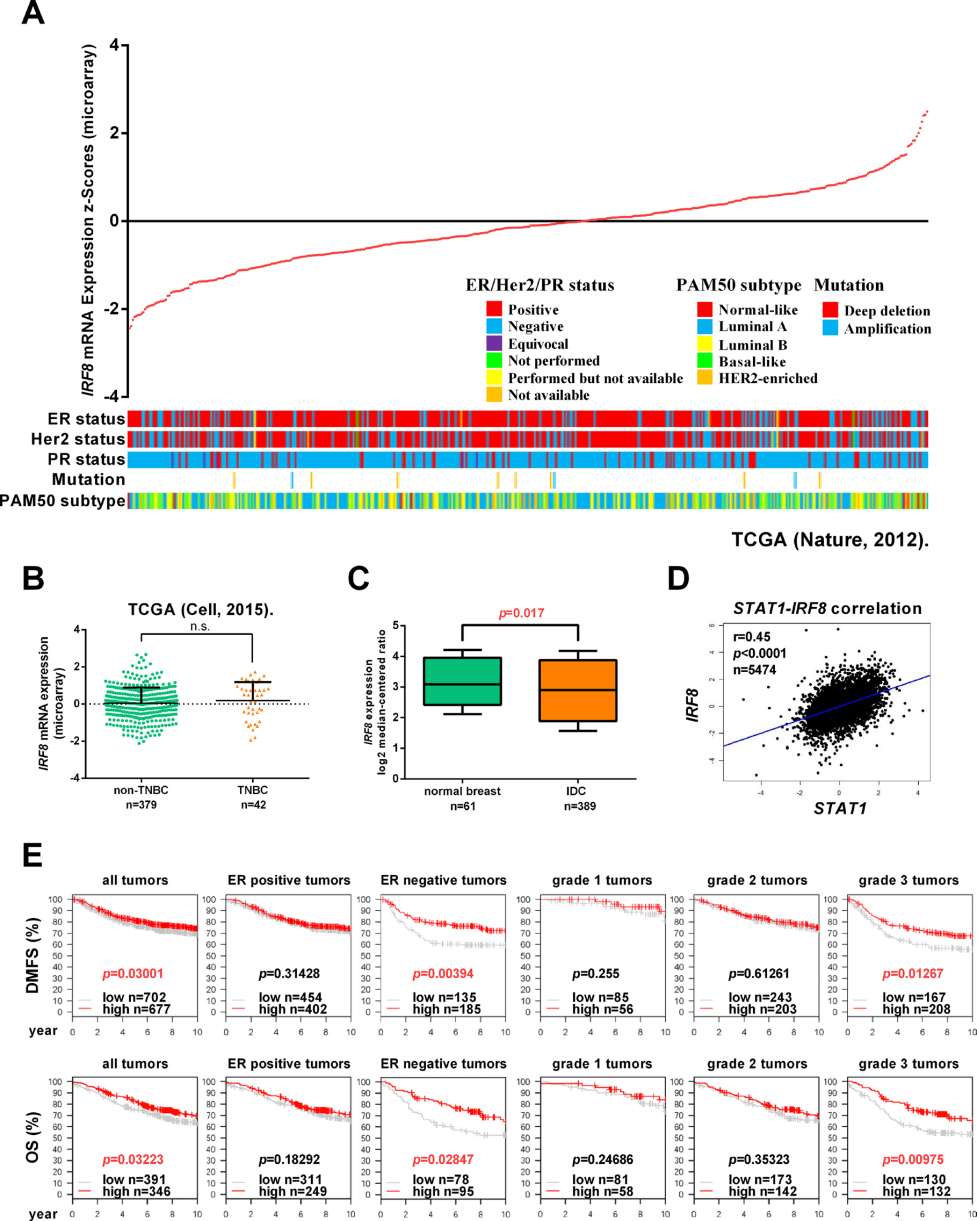
*IRF8* is an independent prognostic factor for breast cancer (**A**) Information on *IRF8* expression was extracted from The Cancer Genome Atlas (TCGA) breast cancer database using cBioPortal online software. Tumors were arranged in ascending order of *IRF8* expression in relation to ER status, PR status, HER2 status, mutation, and PAM50 classification. (**B**) *IRF8* expression was compared between triple-negative breast cancers (TNBCs) (*n* = 379) and non-TNBCs (*n* = 42) in the TCGA breast cancer database (*p >* 0.05). (**C**) *IRF8* expression was compared between normal breast tissues (*n* = 61) and invasive ductal carcinomas (IDC) (*n* = 389) in the TCGA breast cancer database using Oncomine online software (*p* = 0.017). (**D**) The correlation between *STAT1* and *IRF8* expression was analyzed using Breast Cancer Gene-Expression Miner v4.0 (bc-GenExMiner v4.0) (*r* = 0.45, *p <* 0.0001, *n* = 5474). (**E**) Distant-metastasis-free survival (DMFS) was compared between high- and low-*IRF8*-expressing breast tumors (*p* = 0.03001) for ER-positive tumors (*p* = 0.31428), ER-negative tumors (*p* = 0.00394), grade 1 tumors (*p* = 0.255), grade 2 tumors (*p* = 0.61261), and grade 3 tumors (*p* = 0.01267). Overall survival (OS) was compared between high- and low-*IRF8-*expressing breast tumors (*p* = 0.03223) for ER-positive tumors (*p* = 0.18292), ER-negative tumors (*p* = 0.02847), grade 1 tumors (*p* = 0.24686), grade 2 tumors (*p* = 0.35323), and grade 3 tumors (*p* = 0.00975).

To investigate the expression of *IRF8* in molecular subtypes of breast cancer, we analyzed expression data for *IRF8* in The Cancer Genome Atlas 2012 (TCGA) breast cancer database using the online cBioPortal (http://www.cbioportal.org/) [24, 25], classified according to estrogen receptor (ER), progesterone receptor (PR), and human epidermal growth factor receptor 2 (HER-2) statuses, mutation and expression of 50 genes (PAM50) (Figure 1A). There was no significant difference in *IRF8* expression between triple-negative breast cancers (TNBCs) (*n* = 379) and non-TNBCs (*n* = 42) in the 2015 TCGA breast cancer database (*p >* 0.05) (Figure 1B). We also analyzed the expression of *IRF8* using online Oncomine software (https://www.oncomine.org/), and showed that *IRF8* was downregulated in invasive ductal breast cancers (*n* = 389) compared with normal breast tissues (*n* = 61) (*p* = 0.017) (Figure 1C). *IRF8* has been shown to be a downstream target gene of IFNγ–STAT1 signaling [10], and we identified a negative correlation between *STAT1* and *IRF8* expression using bc-GenExMiner v4.0 online software (*n* = 5474, *r* = 0.45, *p <* 0.0001) (Figure 1D) [26, 27]. Importantly, high expression of *IRF8* was associated with relatively high distant-metastasis-free survival (DMFS) (*p* = 0.03001) and overall survival (OS) (*p* = 0.03223) in breast cancers, especially in ER-negative tumors (DMFS, *p* = 0.00394, OS, *p* = 0.02847) and grade 3 tumors (DMFS, *p* = 0.01267, OS, *p* = 0.00975) (Figure 1E). These results suggest that IRF8 may be an independent prognostic factor in breast cancer, especially in patients with ER-negative tumors.

### Promoter methylation contributes to IRF8 downregulation in breast cancer cells

**Figure 2:**
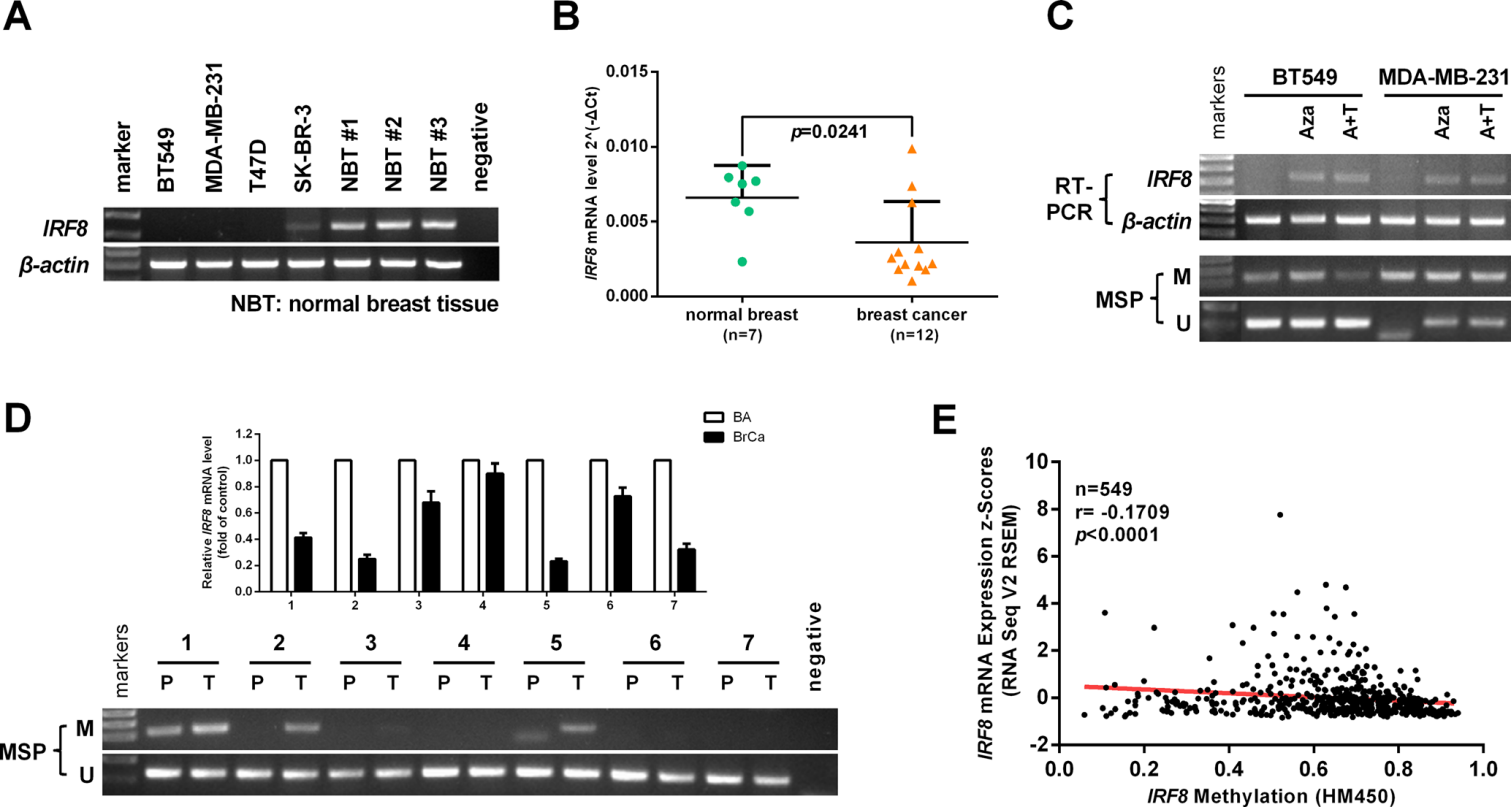
Promoter methylation contributes to *IRF8* downregulation in breast cancer cells (**A**) *IRF8* expression in a panel of human breast cancer cell lines and three normal breast tissue samples was detected by RT-PCR. β-actin was used as a control. (**B**) Expression of *IRF8* in normal breast tissues (*n* = 7) and breast cancer tissues (*n* = 12) detected by real-time PCR, with β-actin as a control. Mean ± SD, *p* = 0.0241. (**C**) The expression and methylation status of *IRF8* were measured in BT549 and MDA-MB-231 cells treated with 5-aza-2′-deoxycytidine (Aza) with or without trichostatin A (TSA) by RT-PCR and methylation-specific PCR (MSP). M: methylated, U: unmethylated. (**D**) Expression and methylation status of *IRF8* in 7 paired breast cancers and matched non-cancerous breast tissues were detected by real-time PCR and MSP, respectively. (**E**) The correlation between *IRF8* methylation and expression in the TCGA breast cancer database was analyzed using cBioPortal online software (*n* = 549, *r* = −0.1709, *p <* 0.0001).

Expression of *IRF8* has been shown to be silenced or downregulated due to promoter hypermethylation in multiple cancers, including breast cancers [11]. We evaluated *IRF8* expression in a panel of breast cancer cell lines and three normal breast tissue samples by RT-PCR. *IRF8* expression was silenced in BT549, MDA-MB-231, and T47D cells, but not in SK-BR-3 cells or normal breast tissues (Figure 2A). In addition, *IRF8* expression was downregulated in primary breast cancers according to real-time PCR (*n* = 12*, p* = 0.0241), compared with adjacent non-cancerous tissues (*n* = 7) (Figure 2B).

Furthermore, *IRF8* expression was reactivated by treatment with 5-aza-2′-deoxycytidine (Aza) with or without the histone deacetylase inhibitor TSA in BT549 and MDA-MB-231 cells, accompanied by decreased methylation of *IRF8* (Figure 2C). We also evaluated the expression and promoter methylation of *IRF8* in 7 paired breast cancer tissues and matched non-cancerous adjacent tissues. 42.9% (3/7) tumors had *IRF8* promoter methylation accompanied by decreased expression of *IRF*8 expression (Figure 2D). Similarly, there was a negative correlation between *IRF8* promoter methylation and its expression in TCGA breast cancer database (*n* = 549, *r* = −0.1709, *p <* 0.0001) (Figure 2E).

These results indicated that *IRF8* was downregulated mainly as a result of promoter methylation in breast cancers.

### IRF8 is frequently methylated in primary breast cancer

**Figure 3:**
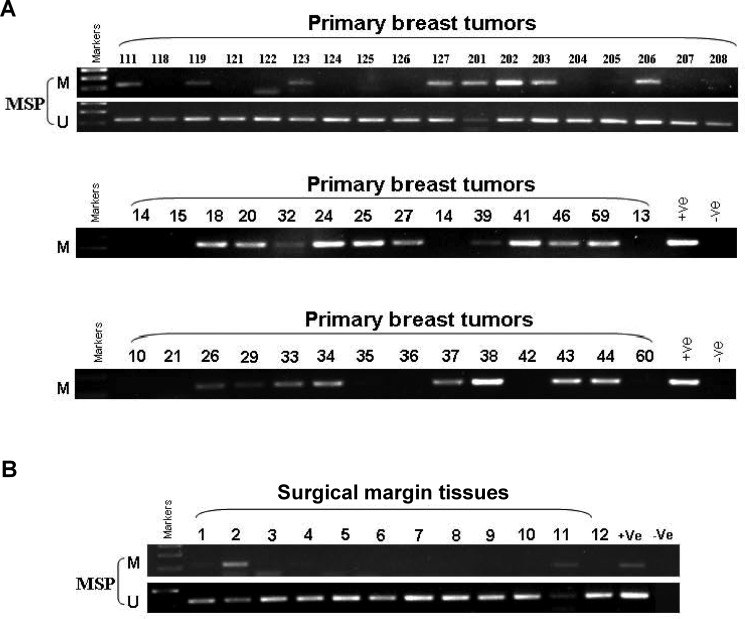
Promoter methylation status of *IRF8* in primary breast cancers Representative analysis of *IRF8* promoter hypermethylation in (**A**) primary breast cancer tissues (*n* = 114) and (**B**) surgical-margin tissues (*n* = 12) in breast cancer patients. M, methylated; U, unmethylated.

It has been demonstrated that *IRF8* is frequently methylated in breast cancers [11]. We therefore determined the *IRF8* promoter methylation status by MSP and analyzed the correlation between methylation status and clinicopathological features in primary breast cancer samples (*n* = 114) and surgical-margin tissues (*n* = 12). Among these patients, the *IRF8* promoter was hypermethylated in 49.12% (56/114) of breast cancer tissues (Figure 3A and Table 1) and 16.67% (2/12) of surgical-margin tissues (Figure 3B and Table 1). However, no correlations were found between *IRF8* promoter methylation and age, tumor size, grade, lymph node metastasis, distant metastasis, ER, PR, or HER-2 (Table 2). Overall, these results demonstrated that the *IRF8* promoter was frequently hypermethylated in breast cancers, but this correlation needs further confirmation in a larger sample group.

**Table 1:** The promoter methylation status of *IRF8* in primary breast cancer-associated tissues

Samples	*IRF8* methylation status		Frequency of methylation
	Methylated	Unmethylated	
**BrCa (*n* = 114)**	**56**	**58**	**49.12% (56/114)**
**BA (*n* = 12)**	**2**	**10**	**16.67% (2/12)**

BrCa, Breast Cancer.

BA, Breast Cancer Adjacent Tissue.

**Table 2:** The correlation between *IRF8* promoter methylation and clinicopathological feature in breast cancers

Clinicopathological features		Numbers	IRF8 methylation status		*P* value
		(*n* = 114)	Methylated	Unmethylated	
Age	≤ 40	15	6	9	0.720
	> 40	88	44	44	
	unknown	11	6	5	
Tumor grade	I	10	4	6	0.629
	II	73	39	34	
	III	6	2	4	
	unknown	25	11	14	
Tumor size	≤ 2.0 cm	35	12	23	0.079
	> 2.0 cm ≤ 5.0 cm	61	36	25	
	> 5.0 cm	7	2	5	
	unknown	11	6	5	
Lymph node metastasis	Positive	48	23	25	0.796
	Negative	54	26	28	
	unknown	12	7	5	
ER status	Positive	55	31	24	0.460
	Negative	35	17	18	
	unknown	24	10	14	
PR status	Positive	41	22	19	0.647
	Negative	49	24	25	
	unknown	24	10	14	
HER2 status	Positive	12	6	6	0.777
	++	41	21	20	
	Negative	36	19	17	
	unknown	25	10	15	

ER, Estrogen Receptor; PR, Progesterone Receptor; HER2, Human Epidermal Growth Factor 2.

Using the χ^2^ tests and Fisher's exact teast.

### IRF8 suppresses cell proliferation *in vitro* and *in vivo*

**Figure 4:**
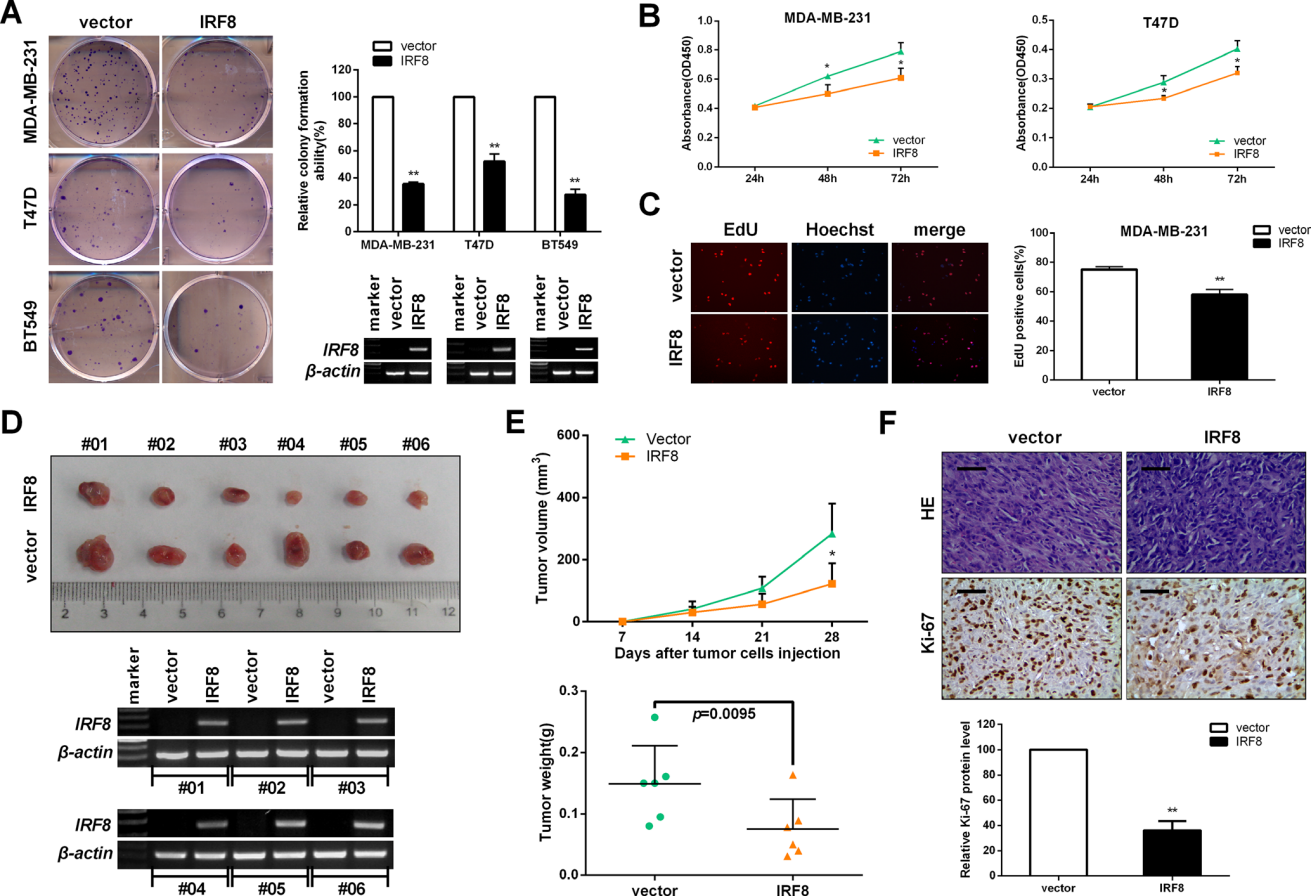
IRF8 suppresses cell proliferation *in vitro* and *in vivo* Cells were transfected with vector and *IRF8* plasmid, and selected with neomycin after 48 h. *IRF8* expression was determined by RT-PCR. Cell proliferation was measured by (**A**) colony-formation assay, (**B**) CCK-8 assay at 24, 48, and 72 h, and (**C**) EdU incorporation assay. Data were based on three independent assays, and representative images are shown. Mean ± SD, *p < 0.05, **p < 0.01. (**D**) Tumors derived from vector- and *IRF8*-expressing MDA-MB-231 cells in mice, each group has 6 mice, and expression of *IRF8* validated by RT-PCR. (**E**) Tumor volume and tumor weight in vector- and *IRF8*-expressing xenografts in MDA-MB-231 cells. Mean ± SD, *p < 0.05. (**F**) Hematoxylin and eosin and Ki-67 staining of *IRF8*-expressing tumors compared with vector-containing tumors. Data were based on three independent assays, and representative images are shown. Mean ± SD, **p < 0.01.

Inactivation of TSGs, including by promoter methylation, is often associated with breast-tumor progression [6]. Several studies have indicated that *IRF8* acts as a functional tumor suppressor by inhibiting cell proliferation [11, 16, 28]. Furthermore, the negative correlation between *IRF8* promoter methylation and its transcriptional level also suggests that *IRF8* might act as a tumor suppressor suppressing promoter methylation in breast cancer. To test this hypothesis, we performed colony-formation assays using MDA-MB-231, T47D, and BT549 cells, CCK-8 assays using MDA-MB-231 and T47D cells, and EdU incorporation using MDA-MB-231 cells. *IRF8* expression was completely silenced by promoter methylation in all these cells [11]. As expected, colony formation (Figure 4A), cell viability (Figure 4B), and EdU incorporation (Figure 4C) were significantly suppressed in *IRF8*-expressed cells compared with control cells.

We further tested the anti-proliferation effect of IRF8 *in vivo*, in tumor-bearing nude mice with MDA-MB-231 xenografts (Figure 4D). Compared with the control (pcDNA3.1) group (*n* = 6), tumor volume and tumor weight were suppressed in the *IRF8*-expression group (*n* = 6) (Figure 4E), accompanied by inhibition of proliferation-related Ki-67 antigen (Figure 4F). These results indicated that IRF8 suppressed cell proliferation of breast cancer *in vitro* and *in vivo*.

### IRF8 induced G2/M cell cycle arrest and apoptosis

**Figure 5:**
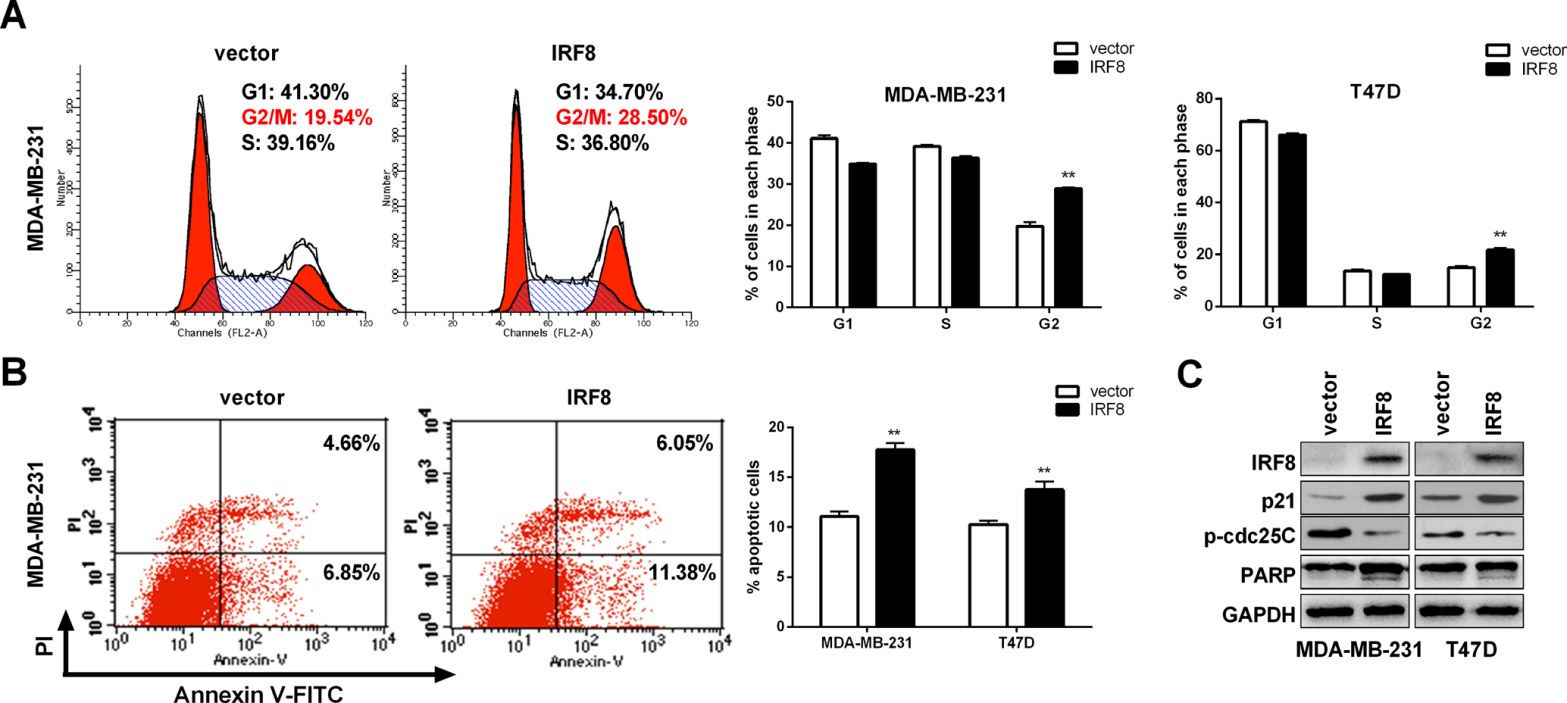
IRF8 induced G2/M cell cycle arrest and apoptosis in MDA-MB-231 and T47D cells Cell cycle distribution was measured in vector- and IRF8-expressing MDA-MB-231 and T47D cells (**A**). Representative flow cytometry plots (left) and histograms of cell cycle alterations (right). The percentage of apoptotic cells was measured in vector- and IRF8-expressing MDA-MB-231 cells. Annexin V-positive cells indicated apoptotic cells. Representative flow cytometry plots (left) and histograms of apoptosis alterations (right). The level of p21, p-cdc25C and cleaved PARP were measured by immunoblotting in vector- and IRF8-expressing MDA-MB-231 and T47D cells. Data were based on three independent assays in MDA-MB-231 and T47D cells, respectively. Mean ± SD, **p < 0.01.

Cell cycle arrest and apoptosis contribute to the inhibition of tumor cell proliferation. Based on existing data in renal cancer [28], we hypothesized that IRF8 might suppress cell growth by modulating the cell cycle and apoptosis. We tested this hypothesis by flow cytometry analysis to determine the cell cycle and apoptosis in vector- or *IRF8*-transfected MDA-MB-231 and T47D cells. The G2/M phase was significantly increased accompanied by decreased S and G0/G1 phases in *IRF8*-transfected cells compared with vector-transfected cells. This effect may have occurred by upregulating p21 and inhibiting p-cdc25C (Figure 5A, 5C). Moreover, the percentage of apoptotic cells was significantly increased in *IRF8*-transfected breast cancer cells by cleaving PARP (Figure 5B, 5C), indicating the pro-apoptotic effect of IRF8.

### IRF8 inhibited breast cancer cell migration and invasion

**Figure 6:**
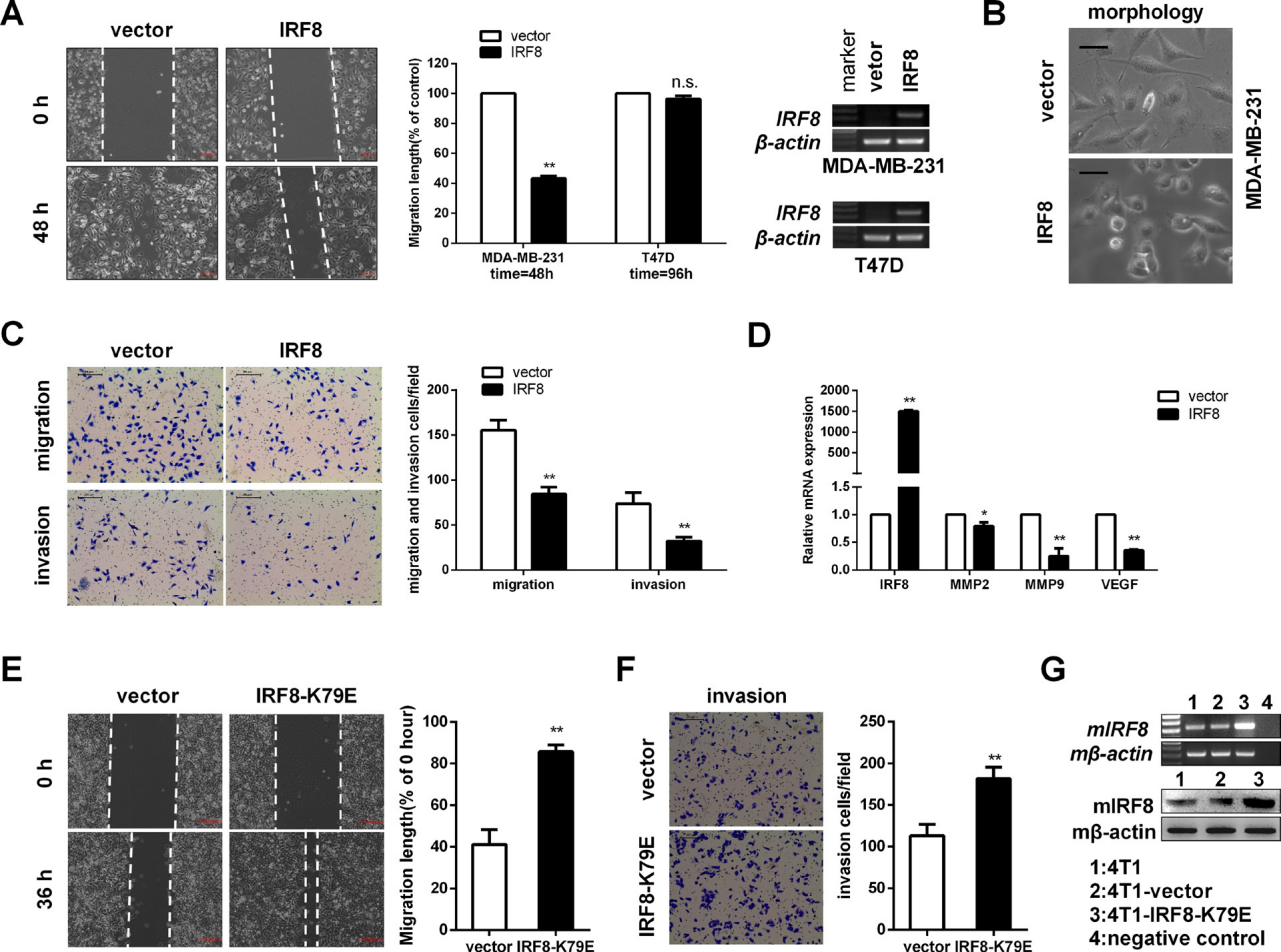
IRF8 suppressed cell migration and invasion in MDA-MB-231 and 4T1 cells *in vitro* (**A**) Wound-healing assay. Confluent monolayers of vector- and *IRF8*-transfected MDA-MB-231 and T47D cells were scarred and migration distance was measured after 48 h and 96 h respectively. Data were based on three independent assays, and representative images are shown. mean ± SD, **p < 0.01. (**B**) Cell morphology of vector- and *IRF8*-expressing MDA-MB-231 cells. Bar = 50 μm. (**C**) Invaded MDA-MB-231 cells in the lower side of the chamber with Matrigel as a barrier were fixed and stained. Numbers of cells were counted by phase-contrast microscopy. Data were based on three independent assays, and representative images are shown. Mean ± SD, **p < 0.01. (**D**) The expression of matrix metalloproteinases (*MMP*) 2 and 9, and vascular endothelial growth factor (*VEGF*) were evaluated under *IRF8* overexpression by real-time PCR, with β-actin as an internal control. Data were based on three independent assays. Mean ± SD, *p < 0.05, **p < 0.01. (**E**) Migration distances of vector- and IRF8-K79E-expressing 4T1 cells after 36 h. Data were based on three independent assays. Mean ± SD, **p < 0.01. (**F**) cell invasion by vector- and IRF8-K79E-expressing 4T1 cells after 24 h. Data were based on three independent assays. Mean ± SD, **p < 0.01. (**G**) IRF8 expression was measured by RT-PCR and immunoblotting in 4T1 cells.

The lung-metastatic potency of tumor cells was previously shown to be enhanced when IRF8 function was disrupted in BALB/c mice [29], indicating that downregulation of *IRF8* expression may contribute to breast cancer cell migration and invasion. To test this hypothesis, we performed wound-healing and Transwell assays to assess the effects of *IRF8* on cell migration and invasion. Wound-healing assays showed that cell migration was significantly inhibited by ectopic *IRF8* expression in MDA-MB-231 cells, but not in T47D cells (Figure 6A). In addition, ectopic *IRF8* expression markedly inhibited MDA-MB-231 cell invasion through a Matrigel barrier, with 10% FBS as an attractant (Figure 6C). This effect may have been mediated by modulating the morphology of MDA-MB-231 cells (Figure 6B), and by downregulating matrix metalloproteinases (*MMP*) 2 and 9, and vascular endothelial growth factor (*VEGF*) (Figure 6D). In contrast, cell migration and invasion were enhanced in cells transfected with IRF8-K79E, which disrupted the function of IRF8 (Figure 6E–6G). These results suggested that the effects of IRF8 on cell migration and invasion may depend on the molecular type of breast cancer, especially in ER-negative tumors.

### IFR8 acts as a downstream target gene of IFN-γ

**Figure 7:**
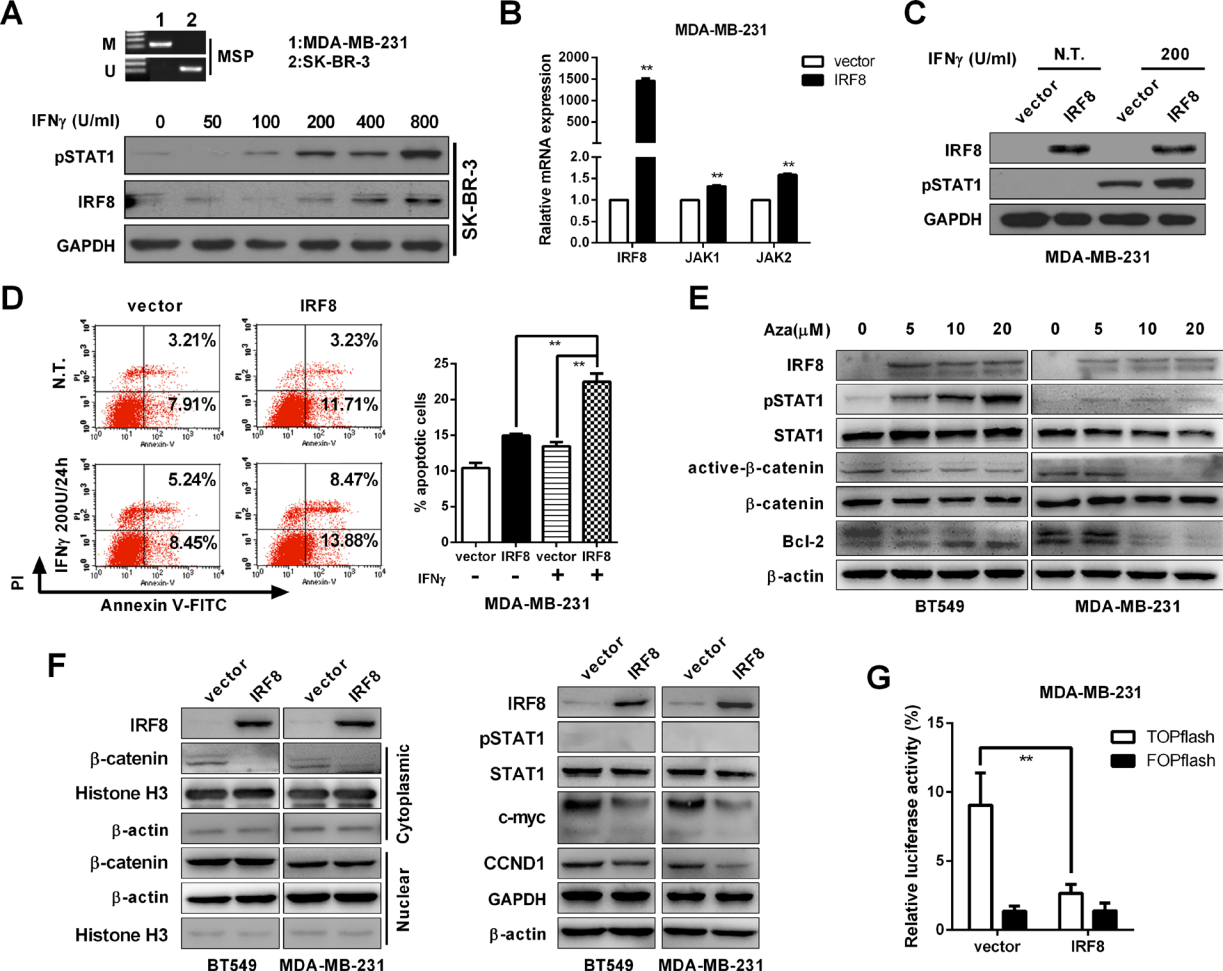
IRF8 expression enhanced the pro-apoptotic effect of IFN-γ and suppressed β-catenin signaling (**A**) Promoter methylation of *IRF8* was measured by MSP in MDA-MB-231 and SK-BR-3 cells. SK-BR-3 cells were treated with different concentrations of IFN-γ (50, 100, 200, 400, 800 U/ml) or vehicle control (DMSO) for 24 h, and the expression levels of pSTAT1 and IRF8 were detected by immunoblotting. (**B**) Expression levels of *JAK1* and *JAK2* were evaluated under *IRF8* overexpression by real-time PCR in MDA-MB-231 cells. Data were based on three independent assays. mean ± SD, **p < 0.01. (**C**) Expression of pSTAT1 was evaluated under IRF8 overexpression with or without IFN-γ by immunoblotting in MDA-MB-231 cells. GAPDH was used as an internal loading control. (**D**) The percentage of apoptotic cells was measured under treatment with IRF8 and/or IFN-γ (200 U/24 h) in MDA-MB-231 cells. Annexin V-positive cells indicated apoptotic cells. Data were based on three independent assays, and representative images are shown. Mean ± SD, **p < 0.01. (**E**) Protein levels of IRF8, pSTAT1, STAT1, active-β-catenin, β-catenin and Bcl2 were measured in BT549 and MDA-MB-231 cells treated with Aza at concentrations of 5, 10, and 20 μM for 3 days. β-actin was used as the control. Data were based on three independent assays, and representative images are shown. (**F**) Effect of ectopic IRF8 expression on β-catenin signaling and its downstream target genes c-myc and CCND1 in BT549 and MDA-MB-231 cells, with GAPDH and β-actin as an internal loading control. (**G**) TOP/FOP luciferase reporter activity assay in vector- and *IRF8*-expressing cells. Data were based on three independent assays. Mean ± SD, **p < 0.01.

*IRF8* acts as a candidate TSG in breast cancer, but its underlying mechanism remains unclear. *IRF8* has been shown to be a downstream target gene of the IFN-γ/STAT1 signaling pathway, and methylation of its promoter can block this inducibility [10, 11]. *IRF8* was induced by IFN-γ treatment via phosphorylation of STAT1 in SK-BR-3 cells (without *IRF8* promoter methylation) (Figure 7A), while restoration of *IRF8* upregulated the expression of JAK1 and JAK2 in MDA-MB-231 cells (Figure 7B), and also enhanced pSTAT1 levels and apoptosis under IFN-γ treatment (Figure 7C, 7D). However, there was no significant difference in pSTAT1 between vector- and *IRF8*-expressing MDA-MB-231 cells (Figure 7F). Moreover, pharmacological demethylation upregulated IRF8 and pSTAT1, and suppressed the levels of Bcl-2 and active-β-catenin (Figure 7E). These data suggest that promoter methylation of *IRF8* disrupts its pro-apoptotic effect in breast cancers.

### Effect of IRF8 on β-catenin signaling in breast cancer

IRF8 has been shown to play an important role in the IFN–γ/STAT1 signaling pathway. However, IRF8, as a transcription factor, may thus modify other signaling pathways, such as β-catenin signaling in breast cancers. The current results showed that ectopic expression of IRF8 suppressed β-catenin activation and downregulated the downstream target genes of β-catenin signaling, including CCND1 and c-myc (Figure 7F). Furthermore, the effect of IRF8 on TcF transcriptional activity was determined by luciferase reporter assay. Luciferase activities were inhibited by *IRF8* in TOPflash, but not in FOPflash, with *Renilla reniformis* luciferase activities as an internal control (Figure 7G).

## DISCUSSION

IRF8 is an IRF with a conserved DNA-binding domain at the N terminal and a IRF-association domain at the C terminal [9]. It can regulate the target gene directly, or as a collaborator through the IFN-stimulated response element, and thus plays an important role in regulating the immune response, and cell growth and differentiation [30]. Evidence has suggested that *IRF8* acts as a TSG in multiple cancers, including leukemia, renal cancer, and soft tissue sarcoma, via regulating cell proliferation, apoptosis, cell cycle distribution, cell invasion, and metastasis [16, 17, 28]. In contrast, IRF8 also has been shown to promote HL-60 proliferation via the transforming growth factor (TGF)-β receptor/TAK-1/p38 pathway [18], and enhance cell motility and invasion by repressing TGF-β signaling in U2OS cells [19], as well as acting as an independent adverse factor in acute myeloid leukemia [31]. However, the role and underlying mechanism of IRF8 in breast cancer remains unclear. In this study, we demonstrated that *IRF8* was downregulated in breast cancers, mainly as a result of promoter hypermethylation. We also showed that *IRF8* may act as a candidate TSG by inducing cell cycle arrest and apoptosis, and inhibiting cell migration and invasion in breast cancer by inhibiting β-catenin signaling. Meanwhile, *IRF8* also act as a downstream target gene of IFN-γ/STAT1 signaling and enhances the pro-apoptotic effect of IFN-γ by regulating STAT1 phosphorylation.

The expression of *IRF8* was downregulated or silenced in breast cancer cell lines and primary breast cancers due to promoter hypermethylation. Furthermore, its expression may be an independent factor for DMFS and OS in ER-negative and grade 3 tumors. It has been reported that 13 genes were hypermethylated in hormone receptor (HR)+, luminal A, or p53 wild-type breast cancers, while 9 genes were hypermethylated in HR−, basal-like, or p53 mutant tumors, indicating differences in DNA methylation patterns among breast cancer subtypes [32]. However, it seems there was no obviously correlation between ER status and the expression of *IRF8*, and there was no significant difference in *IRF8* expression between ER-positive (*n* = 323) and ER-negative (*n* = 92) tumors (data not shown), or between TNBCs and non-TNBCs. In this study, the *IRF8* promoter was methylated in 49.12% (56/114) of breast cancers, which was slightly higher than in breast cancers in previous studies (36%, 5/14) [11]. Moreover, our results were in line with several other cancers, including multiple myeloma, nasopharyngeal, esophageal, lung, and renal cancers [11, 12, 15, 28]. Notably, *IRF8* methylation was associated with tumor grade in renal cancer [28]. However, the current study found no correlation between *IRF8* methylation and clinicopathological features, including ER status, based on 114 breast cancer cases. Further studies with more patients, especially with ER-negative tumors, and more detailed follow-up information may be necessary to clarify this relationship.

Previous studies reported that *IRF8* acted as a TSG in solid and non-solid tumors [15, 28]. Our data showed that IRF8 performed as a candidate tumor suppressor by inducing G2/M phase cell cycle arrest and apoptosis in MDA-MB-231 and T47D cells, consistent with the function of IRF8 in renal cancer [28], and also by inhibiting cell migration and invasion in MDA-MB-231, but not in T47D cells. The effects of IRF8 thus seemed to be more pronounced in ER-negative breast cancer cells, supporting the prognostic effect of *IRF8* expression in patients with ER-negative breast cancers.

*IRF8* has previously been shown to be an inducible gene of IFN-γ/STAT1 signaling, and its promoter methylation disrupted the IFN-γ response [10, 11]. In this study, *IRF8* was induced by IFN-γ in SK-BR-3 cells (without *IRF8* promoter methylation) but not in MDA-MB-231 cells (with *IRF8* promoter methylation) (data not shown). In addition, we showed that IRF8 enhanced the anti-tumor activity of IFN-γ in MDA-MB-231 cells, possibly via upregulated expression *JAK1* and *JAK2*. Meanwhile, pSTAT1 was not induced by ectopic *IRF8* in the absence of IFN-γ. However, accumulated data have shown that IFN-γ-induced genes, such as *STAT1*, promote tumor growth, metastasis, and resistance to therapy. The synergistic effect between IFN-γ and demethylating agents in cancer treatment may thus need further study [33].

IRF8, as a transcription factor, also exerts its effect by modulating the downstream target gene and/or signal pathway directly. Oncogenes (*YAP1*, *Survivin*) were shown to be repressed and TSGs (*p21*, *PTEN*, *CASP1*) upregulated under *IRF8* overexpression in renal cancer [28], and negative feedback occurred between IRF8 and β-catenin in leukemia [23]. The current study revealed a relationship between IRF8 and β-catenin, and indicated that β-catenin signaling was suppressed by ectopic expression of *IRF8*, followed by decreased luciferase activities and downregulation of CCND1 and c-myc. Thus, β-catenin signaling was implicated in the anti-tumor effect of IRF8 in multiple cancers, including breast cancers.

In conclusion, expression of the IFN-γ-inducible gene *IRF8* may be downregulated due to promoter methylation, impairing its anti-tumor effect by modulating β-catenin signaling in breast cancers.

## MATERIALS AND METHODS

### Primary breast samples

Primary breast-associated tissues were obtained from the Department of Endocrine and Breast Surgery, the First Affiliated Hospital of Chongqing Medical University, P.R. China, from January 2013 to October 2014, stored at −80°C in Chongqing Key Laboratory of Molecular Oncology and Epigenetics, the First Affiliated Hospital of Chongqing Medical University, Chongqing, China, and confirmed by pathology. None of the patients had received preoperative anti-tumor treatment, and written informed consent was obtained from each patient before surgery. This research was approved by the Institute Ethics Committee of the First Affiliated Hospital of Chongqing Medical University.

### Cell culture and transfection

Four cell lines were used in this study, including BT549, MDA-MB-231, SK-BR-3 and T47D [34]. All cells were cultured in RPMI 1640 or DMEM (Gibco-BRL, Grand Island, NY, USA), supplemented with 10% fetal bovine serum (Gibco-BRL), and 100 U/ml penicillin–streptomycin (Gibco-BRL), and maintained at 37°C with 5% CO_2_. All transfections were performed using Lipofectamine 2000 (Invitrogen, Carlsbad, CA, USA) following the manufacturer's instructions. pcDNA3.1, pcDNA3.1-*IRF8* and pcDNA3.1-*IRF8*-K79E plasmids were transfected into cells at a concentration of 4 μg, and selected by neomycin at 48 h after transfection.

### RNA and DNA extraction

Total RNA and genomic DNA were extracted using TRIzol reagent (Invitrogen) and a QIAamp DNA Mini Kit (Qiagen, Duesseldorf, Germany), respectively, as described previously [35]. Their concentrations were measured with a NanoDrop 2000 spectrophotometer (Thermo Fisher Scientific, Waltham, MA, USA), and their levels were determined by gel electrophoresis.

### Reverse transcription, semi-quantitative and quantitative polymerase chain reaction

Reverse transcription, semi-quantitative polymerase chain reaction (PCR) was performed as described previously, using Go-Taq polymerase (Promega, Madison, WI, USA) [11]. Quantitative PCR was performed using a SYBR^®^ Green PCR Master Mix kit (Invitrogen) and an Applied Biosystem 7500 Real-time PCR System (Applied Biosystems, Foster City, CA, USA). β-actin served as a control. The relative expression of *IRF8* was evaluated using the 2^(−ΔCt)^ method. All assays were performed three times, independently. Primers are listed in Supplementary Table 1.

### Bisulfite treatment and methylation-specific PCR

Bisulfite treatment and methylation-specific PCR (MSP) were performed as described previously [11, 36, 37]. Briefly, bisulfite-treated DNA was amplified to evaluate the methylation status of *IRF8* by MSP with primers *IRF8*-m1 and *IRF8*-m2 to detect methylated DNA, and *IRF8*-u1 and *IRF8*-u2 to detect unmethylated DNA. Primers are listed in Supplementary Table 1.

### Colony-formation assay

Anchorage-dependent growth was evaluated by colony-formation assay. IRF8-expressing cells and control (pcDNA3.1) cells (200, 500, or 1000) were re-plated in six-well plates with the indicated concentrations of neomycin. Surviving colonies (>50 cells) were counted after 2 weeks following fixation and staining. All experiments were performed three times, independently.

### Cell-viability assay

Cell viability was evaluated using a CCK-8 kit (Beyotime Institute of Biotechnology, Jiangsu, China). Briefly, *IRF8*-expressing cells and control (pcDNA3.1) cells (MDA-MB-231 and T47D) were seeded in 96-well plates after transfection, and cell viability was measured with the CCK-8 kit at 24, 48, and 72 h. All experiments were performed three times, independently.

### 5-Ethynyl-2′-deoxyuridine assay

Cell proliferation was detected using 5-ethynyl-2′-deoxyuridine (EdU) kits (RiboBio, Guangzhou, China). Briefly, cell proliferation was measured by analyzing EdU incorporation during DNA synthesis after transfection. All assays were performed three times, independently.

### Wound-healing and Transwell assays

Cell migration and invasion ability were evaluated by wound-healing and Transwell assays, respectively. Briefly, *IRF8*- or *IRF8*-K79E-expressing cells and control (pcDNA3.1) cells were plated in six-well plates, and wounded using sterile tips once confluent. Cell migration was semi-quantified by measuring the migration distance under phase-contrast microscopy (Leica DMI4000B, Milton Keynes, Buckinghamshire, UK). Transwell chambers (Corning Life Sciences, NY, USA) with a pore size of 8 μm were used to evaluate cell migration and cell invasion, with a Matrigel (BD Biosciences, San Jose, CA, USA) barrier. Cells in the lower side of the chamber were counted after fixation and staining under phase-contrast microscopy (Leica). All assays were performed three times, independently

### Flow cytometry analysis

Cell cycle distribution and the percentage of apoptotic cells were analyzed by flow cytometry [38]. Briefly, cells were stained with propidium iodide (PI) to analyze cell cycle, after transfection and fixation. Cells were double stained with annexin V–fluorescein isothiocyanate/PI to detect apoptosis. All assays were evaluated using a Cell Quest kit (BD Biosciences). All assays were performed three times, independently.

### Immunoblotting

Immunoblotting was performed as described previously [39]. Briefly, whole cells were lysed in RIPA lysis buffer (Beyotime Institute of Biotechnology) with a protease inhibitor cocktail (Pierce, Cramlington, UK). Lysates were separated by 10%–12% sodium dodecyl sulfate-polyacrylamide gel electrophoresis and transferred onto polyvinylidene fluoride membranes (Merck Millipore, Billerica, MA, USA), and incubated overnight at 4°C with the following primary antibodies: IRF8 (Santa Cruz Biotechnology, CA, USA), p21, p-cdc25C, poly (ADP-ribose) polymerase (PARP), pSTAT1, STAT1, β-catenin, β-actin, Bcl-2, Histone H3 (Cell Signaling Technology, Danvers, MA, USA), active-β-catenin (Merck Millipore), CCND1, c-myc, GAPDH (Epitomics, Burlingame, CA, USA), followed by incubation with secondary antibody. The bands were visualized using ECL Plus Detection Reagents (RPN2132; GE Healthcare Life Science, Buckinghamshire, UK). All assays were performed three times, independently.

### *In vivo* tumor model

The anti-tumor function of the target gene was evaluated using an *in vivo* model. Vector- and IRF8-expressing MDA-MB-231 cells (5×10^6^) were injected subcutaneously into nude mice (*n* = 6 in each group). The tumor volumes were monitored (volume = 0.5 × length × width^2^ per week), and the weights of the xenografts were measured after sacrifice. All procedures for constructing the tumor model were approved by the Institute Ethics Committee of the First Affiliated Hospital of Chongqing Medical University.

### Immunohistochemistry

Standard streptavidin–peroxidase immuno histochemistry was performed using an UltraSensitive TM SP Kit (Maixin-Bio, Fujian, China) according to the manufacturer's instructions. Sections were dewaxed, rehydrated, and blocked, and then incubated with a primary antibody against Ki-67 (1:50 dilution). The sections were then treated with a secondary antibody and stained with diaminobenzidine. The staining was assessed by a trained pathologist using Image-Pro Plus (IPP, version 6.0). All assays were performed three times, independently.

### Dual-luciferase reporter assay

The effect of IRF8 on TcF transcriptional activities was determined by luciferase reporter assay [40]. Briefly, the TcF-responsive luciferase construct TOPflash or FOPflash (containing a mutant TCF/LEF binding site) was cotransfected with *IRF8* or control vector, with Renilla luciferase reporter pRL-TK aa an internal control (Promega). Luciferase activities were determined 48 h later using a dual-luciferase reporter assay kit (Promega). All assays were performed three times, independently.

### Statistical analysis

Data were analyzed using SPSS 17.0 software (SPSS, Inc., Chicago, IL, USA) and presented as mean ± standard deviation (SD). Two-tailed Student *t*-tests were used to determine *p* values. Correlations between methylation status and clinicopathological features were analyzed using χ^2^ and Fisher's exact tests. *p <* 0.05 was considered significant.

## SUPPLEMENTARY TABLE


