# Supplementary material for: The tumor suppressor interferon regulatory factor 8 inhibits β-catenin signaling in breast cancers, but is frequently silenced by promoter methylation
Source: Oncotarget. 2017 Mar 23;8(30):48875–88. doi: 10.18632/oncotarget.16511 (PMC5564732; doi:10.18632/oncotarget.16511)
Supplement: Supplementary file 1 [file oncotarget-08-48875-s001.pdf]

The tumor suppressor interferon regulatory factor 8 inhibits β-catenin signaling in breast cancers but is frequently silenced by promoter methylation

Supplementary Materials

Supplementary Table 1: PCR primers sequences and reaction conditions

PCR	Primers	Sequence (5′–3′)	Size (bp)	T_A(°C)	cycle
MSP					
	IRF8 M1	ATTTTCGGGGTTGTTCGTTC	120	60	40
	IRF8 M2	CACCTAAAATCCAAAAACAACG			
	IRF8 U1	GTATTTTTGGGGTTGTTTGTTT	126	60	40
	IRF8 U2	CTCACACCTAAAATTCAAAAACAACA			
RT-PCR					
	IRF8 F	TCCGGATCCCTTGGAAACAC	240	55	32
	IRF8 R	CCTCAGGAACAATTCGGTAA			
	β-actin F	TCCTGTGGCATCCACGAAACT	315	55	23
	β-actin R	GAAGCATTTGCGGTGGACGAT			
Real-time PCR					
	IRF8 F	TCCGGATCCCTTGGAAACAC	240	60	40
	IRF8 R	CCTCAGGAACAATTCGGTAA			
	VEGF F	ACTTTCTGCTGTCTTGGGTG	311	60	40
	VEGF R	CTGCATGGTGATGTTGGACT			
	MMP2 F	TTTGACGGTAAGGACGGACTC	346	60	40
	MMP2 R	CCTGGAAGCGGAATGGAA			
	MMP9 F	CCTGGAGACCTGAGAACCAATC	79	60	40
	MMP9 R	CCACCCGAGTGTAACCATAGC			
	JAK1 F	GAGAGTACACAGCAGAGGAA	104	60	40
	JAK1 R	GAGCTTGGTGTTCTCGTCATA			
	JAK2 F	TTTTGACAAGGAAGCGAATA	108	60	40
	JAK2 R	CCTCACCTGAAGGACCAC			
	β-actin F	TCCTGTGGCATCCACGAAACT	315	60	40
	β-actin R	GAAGCATTTGCGGTGGACGAT			

TA , Annealing Temperature.
